# Hypothesis: Ran GTPase-Based Potential Therapeutic Interventions Against Lethal Microbial Infections

**DOI:** 10.1100/tsw.2002.148

**Published:** 2002-03-12

**Authors:** Peter M.C. Wong

**Affiliations:** Department of Pathology and Laboratory Medicine, Fels Institute for Cancer Research and Molecular Biology, Temple University School of Medicine, Philadelphia, PA 19140, USA

**Keywords:** innate immune response, septic shock, gene therapy, immune modulation, immune tolerance, infectious disease, Ran GTPase, adenovirus

## Abstract

Host innate immune response represents a vital immediate defense against infections by a diverse group of microorganisms that include bacteria, viruses, and fungi. Many types of cell surface receptors in mammalian cells specifically recognize particular groups of microorganisms and transmit response signals to the nuclei via multiple signal transduction pathways. These signaling pathways must merge at some point and are likely to be redundant, as the host innate immune response to many microorganisms is remarkably similar; it is characterized by the production of proinflammatory cytokines such as TNF*α*, IL-1, and IL-6 by the principal cell types – macrophages and dendritic cells. Since these cytokines influence greatly the magnitude of the cascade of inflammatory events, the proportion and the actual amount of each among the cytokine group may be a characteristic of each type of infections. Immune modulation by systematically up-regulate or down-modulate these cytokines would conceivably have major therapeutic potential. We have recently shown that two alleles of Ran cDNAs – RanT/n and RanC/d – may possess these characteristics. Thus the applica-tion of Ran to the treatment of septic shock, lethal anthrax shock, or adenovirus-induced toxicities may open up many interesting possibilities in the future.

## INNATE IMMUNITY

Innate immune response to a variety of microbial organisms represents an important first line host defense in limiting infection. Despite major differences in specific actions of the toxins or molecules expressed by each microorganism in causing a particular disease, there is a remarkable uniformity in the host in expressing an immediate and impressive immune response against invading microorganisms. This defense is expressed through the principal cell types —

macrophages and dendritic cells — which are often the principal targets for microbial infections. Once stimulated, these cells produce elevated amounts of proinflammatory cytokines, such as TNF-α, IL-1, and IL-6, which then trigger a cascade of inflammatory reactions, leading to the production of other additional cytokines, chemokines, antimicrobial peptides, nitric oxide, and many other metabolites. The end point of these inflammatory events normally would lead to effective elimination of the invading microorganisms at the end. However, a number of clinical manifestations clearly indicate that excessive host innate inflammatory response could lead to undesirable or even detrimental consequences such as fever, hypotension, septic shock, and death.

To limit infection, cells in the host are constantly challenged with the daunting task of recognizing diverse groups of microbial organisms. Recent advances reveal the presence of specific receptor families such as the TLR (toll-like receptors) and the TREM (triggering receptor expressed in monocytes) that are present on the surface of mammalian cells[1,2,3]. These receptors are able to recognize highly conserved three-dimensional molecular patterns present on molecules of the invading microorganisms, sometimes called PAMP (pathogen associated molecular patterns). Ten or more TLR molecules are found on the surface of mammalian cells, each of which could presumably recognize a distinct group of microbial organisms with specificity defined by the structure of PAMPs. For example, TLR4 can be activated by lipopolysaccharide (LPS) of Gram-negative bacteria, other bacterial PAMPs, and respiratory syncytial virus[4]; TLR2 is involved in mycobacteria and fungi[5,6,7]; and finally, TLR5 can be stimulated by bacterial flagellin[8].

## MULTIPLE SIGNAL TRANSDUCTION PATHWAYS FOR INNATE IMMUNE RESPONSES

The existence of this large family of TLR and perhaps others such as TREM-1 on the surface of mammalian cells to combat a variety of pathogenic microbes strongly suggests that multiple signal transduction pathways are in operation. Merging of these signal transduction pathways at some point downstream of each of these pathways would be consistent with a remarkably uniform host innate inflammatory response — the production of proinflammatory cytokines, such as TNF-α, IL-1, and IL-6 — elicited by stimulated macrophages and dendritic cells in the host, thereby triggering the typical cascade of inflammatory reactions. Much research is needed for the elucidation of each of these pathways, as many of these TLR or TREM molecules have been identified only recently. Clues for multiple pathways merging to a central molecule already exist. For example, LPS from Gram-negative bacteria *E. coli* or *Samonella* has been shown to activate TLR4, whose cytoplasmic domain interacts with MyD88, then in turn, with IRAK, TRAF6, TAK, and then the transcriptional activator NF-κB. The transcriptional factor NF-κB has been shown to be a central molecule for many different signal transduction pathways, leading to the transcriptional activation of TNF-α, IL-1, and IL-6 genes[9].

Most likely due to the important nature of the first line host defense against diverse pathogenic microbial infections, alternative pathways of innate immune response exist and may be redundant by function. Such documentation also exists. CD14, MyD88, and NF-κB are related to one another in LPS endotoxin-mediated signal transduction pathway. However, mice with homozygous deletion of the CD14 gene remain responsive to LPS endotoxin stimulation, albeit the LPS dose required to elicit the biological effects is higher than normally required[10]. Likewise, MyD88-deficient mice exhibit unresponsiveness to LPS but NF-κB activity in these mice remains inducible by LPS, which suggests that a MyD88-independent, endotoxin-mediated signaling is operative in these knockout mice[11]. Recently, TREM-1 has also been shown to play a key role in septic shock and it can initiate a signaling pathway independent of NF-κB, via interaction with DAP12[3]. Importantly, TREM-1 is a member of a super-Ig family and has no homology to the large family of TLRs.

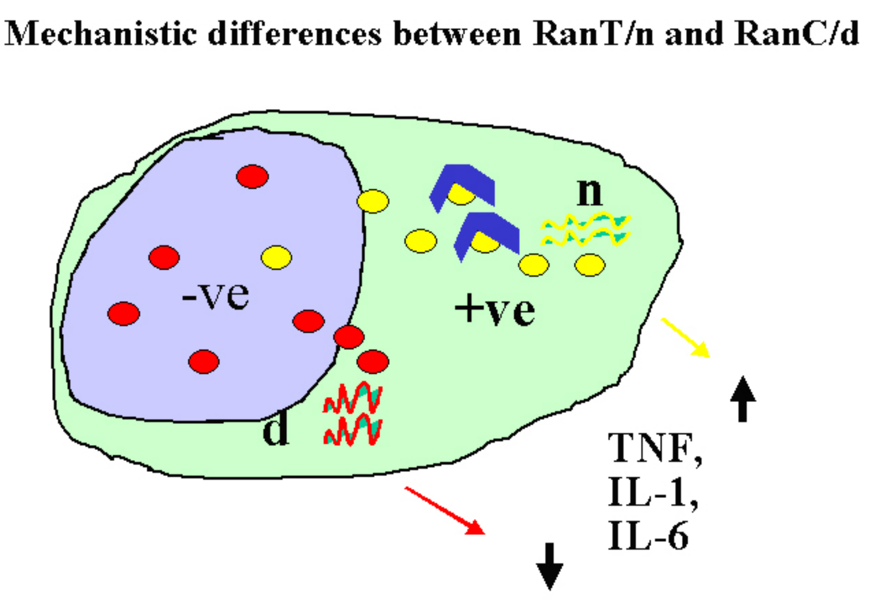

**FIGURE 1**. Schematic illustration of the mechanistic differences between RanT/n and RanC/d. The single point mutation of Ran mRNA leads to different intracellular localization of the two species of Ran mRNA and protein[15]. There is no significant difference in transcriptional and translational rates. The preferred cytoplasmic localization of RanT/n proteins renders them susceptible to enzymatic degradation by proteases located in the cytoplasm. All cytoplasmic Ran proteins transmit a positive signal to activate transcription of proinflammatory cytokine genes; all nuclear Ran proteins produce a negative signal. Thus, RanT/n-transduced cells would produce an overall positive signal for the production of proinflammatory cytokines, whereas for RanC/d-transduced cells, a down-regulation of cytokine production. Yellow wiggly lines and circles, RanT/n mRNAs and proteins, respectively; red wiggly lines and circles, RanC/d mRNAs and proteins; filled blue shape, cytoplasmic protease; +ve or -ve = positive or negative signal transmission for proinflammatory cytokine production. Black up or down arrows = increase or decrease levels of TNF, IL-1, and IL-6.

By cDNA functional expression cloning, we have shown that Ran GTPase is involved in LPS endotoxin response[12]. We further showed that a single point mutation in the 3'-UTR of Ran mRNA results in profound biological changes, which can be applied beneficially to a variety of clinical conditions[13,14,15]. This single point mutation leads to conformational changes of the RanT/n and RanC/d mRNAs, hence the differential amounts of nuclear and cytoplasmic Ran in transduced macrophages. The preferential cytoplasmic localization of RanT/n renders the proteins susceptible to proteolytic enzymes present in the cytoplasm, resulting in reduced accumulation of RanT/n in the nucleus at steady state (Fig. 1).

Ran GTPase, like NF-κB, can be found in both the cytoplasm and in the nucleus, albeit most Ran proteins are present in the nucleus at steady state. Like NF-κB, Ran is also known to be involved in diverse biological functions, which include nuclear transport of macromolecular RNA and proteins, RNA modification, chromosome stabilization, cell cycle progression, and recently, in binding to microtubule spindle formation during mitosis[16]. Unlike NF-κB, both cytoplasmic and nuclear Ran GTPases are known to bind to various distinct groups of proteins involved ion signaling and therefore Ran in each of these intracellular compartments are likely involved in distinct biological functions. Along this light, the differences in the relative endotoxin resistance manifested by RanT/n- and RanC/d-transfected cells correlate with their expected biological phenotypes and their initial preferential intracellular location[15]. Thus the cytoplasmic Ran proteins are involved in transmitting signals for activating or enhancing the transcription of proinflammatory cytokine genes, whereas the nuclear Ran proteins are likely involved in reducing such transcription (Fig. 1).

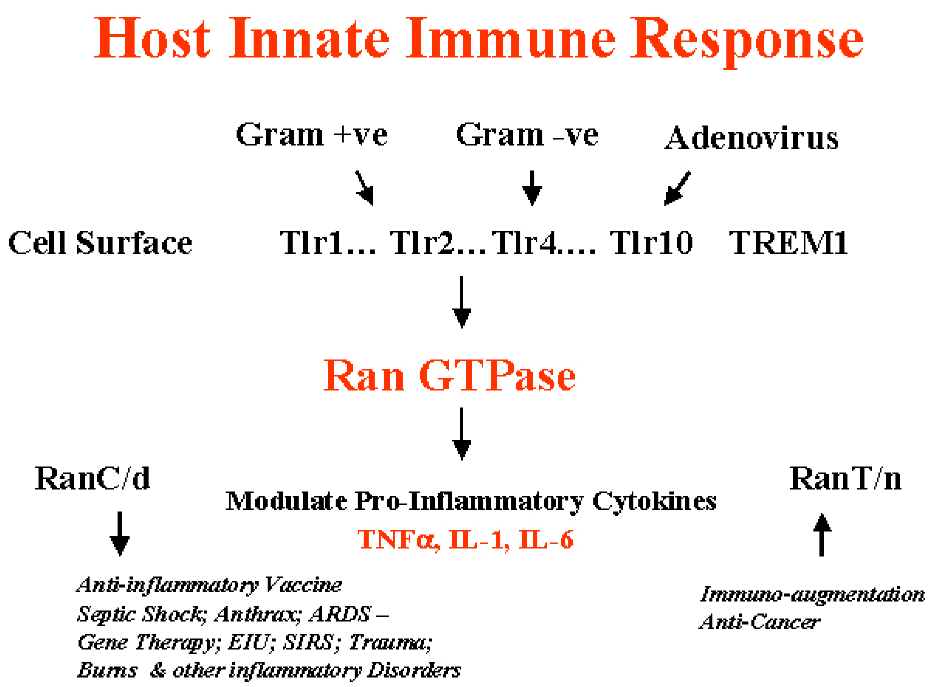

**FIGURE 2.** The role of Ran GTPase in host innate immune response and its potential clinical applications.

## POTENTIAL CLINICAL APPLICATIONS

The discovery that a single point mutation within the 3'-UTR of Ran mRNA can lead to major biological changes provided us an opportunity to uncover the potential value of Ran in clinical medicine. The comparison between RanT/n and RanC/d using a variety of biological assays reveals that RanC/d can potentially be used for immune tolerance whereas RanT/n can be used to augment host immune response (Fig. 2).

Ran can potentially be applied to treating endotoxic shock. Successful gene transfer and expression of RanC/d cDNA in mice confer resistance, and expression of RanT/n in mice confers increased sensitivity to endotoxic shock[17]. Ran can also be applied to treating adenovirus-induced, excessive host innate inflammatory response. Expression of adenovirus antigens in gene therapy clinical trials using adenoviral vectors has deleterious effects on patients[18]. These effects are primarily due to an excessive host innate inflammatory response against viral antigens on either the transduced macrophages or dendritic cells, or on the adenovirus infused in the host[19,20,21]. Our recent studies have shown that RanC/d could protect sensitive mice against adenovirus-induced innate immune responses, and that this protection is related to a reduced serum level of IL-6 and TNF-α[22]. By contrast, expression of RanT/n rendered sensitive mice even more sensitive to adenoviral antigen stimulation, again emphasizing the significance of this single base change at the 3'-UTR of the mRNA.

These preclinical animal data are consistent with the fact that the biological "potency" of Ran GTPase in endotoxin stimulation is moderate and, for this reason, Ran is likely involved in an alternate pathway of endotoxin-mediated signal transduction. They also suggest that Ran is involved in signaling pathways other than one mediated by endotoxin, such as those stimulated by adenovirus as well. As Ran is involved in more than one signaling pathway and its moderate biological potency in any particular one, it may also play a role in host innate immune response induced by Gram-positive bacteria, such as *Bacillus anthracis*. Septic shock manifested in patients with anthrax also exhibits high elevation of serum IL-1 and TNF-α, triggering the typical cascade of inflammatory response[23,24,25]. A moderate down-regulation of proinflammatory cytokine production can have a very significant clinical outcome, as these cytokines are the initiators of the cascade of inflammatory events. In this way, the host would remain immunocompetent against the infections but might also avoid the development of excessive innate inflammatory response leading to deleterious effects such as shock and death.

The ability of RanC/d to down-modulate the production of more than one proinflammatory cytokine in a dominant negative fashion in key target cells would represent a new treatment modality[14,22]. The rationale for it is that despite different modes of action of each product from a variety of microbial organisms, the host innate immune response against the microbial organisms is remarkably similar, yet the relative proportion of each of a set of proinflammatory cytokines, and thus the type of lethal shock seen in the host, is a reflection of a particular type of microbial infection. For example, elevation of TNF-α is the primary but not the only proinflammatory cytokine observed in Gram-negative endotoxic shock[26], elevation of IL-1 is readily observed in patients with septic shock induced by *Bacillus anthracis*[23,24], and elevation of serum IL-6 appears to be prominent in shock induced by adenoviral antigens[18,19,20]. As Ran is involved in more than one type of innate immune responses, the ability of RanC/d to down-modulate these proinflammatory cytokines across the board would present itself a very likely candidate to which a focused research on it would lead to successfully treating patients vulnerable to lethal shocks induced by a variety of microbial infections.

Conversely, RanT/n may be applicable to clinical situations where augmentation of innate immune response could be beneficial to the patients, as in plaque developed as a result of infection by *Yersinia pestis*, or in cancer. The initial phase of patients with plague is characterized by a strong suppression of inflammatory responses, due to suppression of NF-κB activity by the Yops proteins of *Yersinia*, hence TNF-α production by the infected macrophages[27]. Augmentation of an alternative pathway of TNF-α stimulation, such as one by RanT/n, in combination with antibiotic treatment, would be an exciting new avenue of therapeutic intervention.

## ACKNOWLEDGEMENT

The author wishes to thank many coworkers in his and collaborating laboratories who have made major contributions to the findings described in this review. Current work described in the author's laboratory has been supported by NIH grants RO1CA70854, RO1AI39159 and RO1AI45951.
